# Economic Policy Uncertainty, Social Development, Political Regimes and Environmental Quality

**DOI:** 10.3390/ijerph19042450

**Published:** 2022-02-21

**Authors:** Hang Su, Yong Geng, Xi-Qiang Xia, Quan-Jing Wang

**Affiliations:** 1 School of Economics and Finance, Xi’an Jiaotong University, 74 Yanta Road, Yanta District, Xi’an 710061, China; tg199333@126.com; 2 School of Business, Shanghai Jiaotong University, 800 Dongchuan Road, Minhang District, Shanghai 200240, China; ygeng@sjtu.edu.cn; 3 School of Business, Zhengzhou University, 100 Kexue Road, Zhengzhou 450001, China; xqxia@zzu.edu.cn

**Keywords:** economic policy uncertainty, environmental performance, increasing EPU, social development, different political regime

## Abstract

This paper aims to examine the influence of Economic Policy Uncertainty (EPU) on environmental performance, as well as the moderating effect of social development and the political regimes in EPU’s influence on environmental performance. To investigate such essential issues, we conducted Generalized Method of Moments (GMM) estimations by utilizing cross-country data covering 137 countries during the period of 2001–2018, according to the Stochastic Impacts by Regression on Population, Affluence and Technology (STIRPAT) model. Our empirical estimations support that EPU negatively affects environmental performance; this idea was still supported when we conducted an empirical analysis by changing the measurements, employing alternative estimations and constructing new samples. Furthermore, not only would the absolute level of EPU bring worse environmental performance, but so would an increase in EPU. Moreover, higher economic performance, globalization and a high quality of governance can help countries to alleviate the adverse environmental effect of EPU. Additionally, EPU’s negative effect on environmental performance is stronger in right-wing countries, autocracies and non-OECD countries, compared to their counterparts. Our study provides substantial policy implications for governments participating in the international treaties of environmental protection, to mitigate environmental degradation.

## 1. Introduction 

With growing social problems such as human diseases, extreme climate events, and natural disasters caused by serious environmental degradation (Kompas et al., 2018; Shahbaz et al., 2019; Coskuner et al., 2020) [1,2,3], it is generally accepted worldwide that protecting the environment is essential for the survival of humans and national sustainable development (Hambira et al., 2020; Huo et al., 2020) [4,5]. Following this idea, many scholars have investigated how to achieve better environmental performance, and what factors can affect nature from the perspective of economic activities, technological innovation, international trade, energy efficiency, energy structure, political regimes, and so on (Khan et al., 2019; Mahadevan and Sun, 2020; Wang et al., 2021b) [6,7,8]. Recently, attributed to the outbreak of coronavirus and turbulent international circumstances, economic policy uncertainty (EPU) for every country experienced a quick change (Baker et al., 2020; Jordà et al., 2020; Bakas and Triantafyllou, 2020) [9,10,11]. EPU can not only change the economic activities or the concerns of policymakers (Balcilar et al., 2016; Degiannakis et al., 2018; Hailemariam et al., 2019; Phan et al., 2019) [12,13,14,15], but can also change firms’ behaviors or decisions toward environmental protection (Guidolin and La Ferrara, 2010; Kang et al., 2017; Olanipekun et al., 2019; Akron et al., 2020) [16,17,18,19], since economic activity and firms’ production methods contribute to environmental degradation (Salahuddin et al., 2018; Shahbaz et al., 2019) [2,20]; thus it is necessary to query whether increasing EPU can affect the environmental performance (Jiang et al., 2019; Yu et al., 2021; Adams et al., 2020) [21,22,23]. 

EPU may affect environmental performance through the following channels: Jiang et al., (2019) [21] supported that EPU can change environmental performance directly through policy, as well as indirectly through economic activity. Firstly, once EPU increases, more attention from policymakers would turn to how to maintain economic growth by stimulating economic activity, thus reducing the importance of environmental protection during the implementation of policies. This may bring about bad regulations on environmental damage and less effort to protect the environment (Amin and Dogan, 2021) [24], as well as a change in the producing decisions of economic entities; the latter is supported by Yu et al. (2021) [22], who stated that firms often use cheap fossil fuels, which generate more environmental pollution while EPU increases. Secondly, EPU can affect environmental performance by influencing economic demand. As EPU increases, economic activity would experience a decrease, thus reducing the economic demand on energy consumption, which may contribute to the improvement of environmental performance (Wang et al., 2020) [25].

However, the second channel which supports that EPU may reduce environmental damage does not consider energy efficiency of energy intensity, which is critical to carbon emissions. Thirdly, we prefer to consider that EPU can affect environmental performance by changing energy intensity (Pirgaip & Dincergok, 2020) [26]. As Al-Thaqeb and Algharabali (2019) [27] proposed, EPU may cause asymmetric effects, since it is hard to predict future policies; there may be unforeseen effects on investment in clean energy or energy-saving technologies. Bloom (2014) [28] showed that the investors often choose more conservative policies with the growing of EPU, thus reducing the R&D activities aiming to increase energy efficiency (Atsu and Adams, 2021) [29]. Based on such channels, EPU causes a rise in energy intensity and motivates corporations to use environmentally ufriendly technologies, which may eventually lead to worse environmental performance. Thirdly, EPU may affect environmental performance by changing the nexus between the energy and the environment. For instance, Ulucak and Khan (2020) [30] declared that EPU can strengthen the negative effect of energy consumption or energy intensity on carbon emissions; furthermore, Chu and Le (2021) [31] argued that EPU has a moderating effect on the impact of clean energy or economic complexity on environmental performance. 

This scenario naturally motivates us query such interesting issues as the following which need to be studied: Firstly, what effect does EPU exert on total environmental performance? Secondly, can increasing or slowing EPU affect environmental performance under the turbulent international circumstances? Thirdly, can the social circumstances of one country change the effect of EPU on environmental performance? Finally, does EPU’s influence on environmental performance vary among different political regimes? Based on our earlier analyses and their main purposes, we propose such following hypotheses:

**Hypothesis** **1 (H1).** *Economic policy uncertainty negatively affects environmental performance*.

**Hypothesis** **2 (H2).** *Social development and political regimes would change the impact of EPU on environmental performance*.

Comparing to previous works of literature, the potential contributions of this work can be seen as follows: Firstly, we carry out an empirical estimation based on multinational panel data for 137 countries; this can be conducted thanks to the World Uncertainty Index for EPU provided by Ahir et al. (2018) [32], which can offer more common findings on EPU’s influence on environmental performance (Chen et al., 2021) [33]. In addition, considering the highly frequent external shock and turbulent international environment, we further take the increase of EPU into account, to study whether increasing or slowing uncertainty also changes environmental performance; this approach is novel among existing articles.

In addition, we further explore the question of whether EPU’s influence on environmental performance varies among different fundamental countries from the perspective of social development, such as the stage of economic development, economic performance, globalization, and quality of governance; answering this question can link EPU with social circumstances, as well as understand, in detail, the impact of EPU on environmental performance within specific societies (Shabir et al., 2021) [34]. Finally, aside from social indicators, we also attach importance to the moderating effect of political regimes from the perspective of democratic or autocratic regimes, as well as left- or right- wing countries; this can offer more exact results for determining the interactive effect of political regimes and EPU in environmental performance. 

The rest of this paper is structured in line with previous empirical studies. Specifically, Section 2 reviews the relevant literature; Section 3 is mainly about the variables, data and estimation method; Section 4 offers the main results and discussions; and Section 5 gives a brief summary of our empirical results and, hence, proposes some suggestions to policymakers.

## 2. Literature Review

While we review the existing studies, we find that limited works investigate the influence of EPU on total environmental performance, with the most relevant ones focusing on the impact of EPU on carbon emissions. Some studies argue that EPU brings about more CO_2_ emissions, while other scholars hold that there exists a negative influence of EPU on CO_2_ emissions. 

Vast amounts of literature propose that EPU causes more CO_2_ emissions, and some studies propose such an idea according to empirical investigation of one specific country (Ulucak and Khan, 2020; Sohail et al., 2021) [30,35]. For instance, Jiang et al. (2019) [21] utilized data for the United States to empirically investigate the influence of EPU on CO_2_ emissions by employing the novel parametric test of Granger causality, and supported that EPU often brings about more CO_2_ emissions, regardless of whether the economic growth is higher or lower. The positive effect of EPU on CO_2_ emissions in the USA is supported by the research of Wang et al. (2020) [25]. Similarly, Adedoyin and Zakari (2020) [36] utilized data for the United Kingdom, which provided a good sample to study uncertainty since it started phasing out of the European Union from 2016, by employing ARDL estimation; the empirical result based on the data from 1985 to 2017 suggested that while EPU reduced carbon emissions in the short run, it would bring about more carbon emissions in the long run. Recently, Yu et al. (2021) [22] utilized the firm-level data of Chinese firms to examine the influence of EPU on CO_2_ emissions, and supported that while the firms experience EPU, their CO_2_ emissions would be increased due to the firms preference for utilizing cheap fossil fuels, which may cause more air pollution. 

Other scholars confirmed the positive effect of CO_2_ emissions according to empirical tests, by using panel data covering multinational countries (Adedoyin and Zakari, 2020; Atsu and Adams, 2021; Amin and Dogan, 2021) [24,29,36]. Adams et al. (2020) [23] examined the role of EPU on carbon emissions by employing the cross-country data for resource-rich countries during 1996–2017 via the estimation of pooled mean group autoregressive distributed lag model; their result supported that EPU not only positively affected carbon emissions in the short run, but also exerted a significantly positive effect on carbon emissions in the long run. Pirgaip and Dincergok (2020) [26] investigated the question of whether there exists causality between EPU and carbon emissions, by conducting a panel Granger causality estimation based on the data covering G7 countries from 1998 to 2018; their results confirmed that the relationship between EPU and carbon emissions varies among such countries. There exists a unidirectional causal link from EPU to carbon emission in United States, Germany and Canada, while a bidirectional causal link between carbon emissions and Environmental Performance Index (EPI) was confirmed in Italy. Similarly, Shabir et al. (2021) [34] examined the impact of EPU on CO_2_ emissions by utilizing the data for 24 developed countries and developing countries during 2001–2019; their results supported that EPU does some harm to environmental quality. Aside from the index of EPU developed by Baker et al. (2016) [37], Adams et al. (2020) [23] utilized the countries at high geopolitical risk as the sample to study the influence of EPU on CO_2_ emissions, by employing the indicator of the World Uncertainty Index (WUI) provided by Ahir et al. (2018) [32] to capture EPU; their results also supported that higher WUI would lead to more CO_2_ emissions. Recently, Atsu and Adams (2021) [29] also measured EPU by utilizing the WUI index to investigate the effect of EPU on CO_2_ emissions, and offered a similar conclusion. 

On the contrary, some investigations argued that EPU would negatively affect carbon emissions; for instance, Chen et al. (2021) [33] conducted a panel estimation with a fixed effect or random effect, based on the data for 15 countries from 1997 to 2019. Their result indicates that there exists a negative influence of EPU on carbon emissions; in other words, EPU tends to be better for environmental performance. Moreover, Doğan and Güler (2021) [38] empirically studied the relationship between EPU and carbon emissions by employing Parks–Kmenta estimation and panel data for G7 countries from 1997 to 2015; the result suggested that EPU would contribute to a reduction in the carbon emissions. Similarly, Liu and Zhang (2021) [39] proposed that EPU has a negative effect on CO_2_ emissions in the easter region of China, by utilizing the data for China from 2003 to 2017 to empirically examine the relationship between EPU and CO_2_ emissions.

Aside from these two ideas, a few scholars support that there exists no significant influence from EPU to carbon emissions. For example, Abbasi and Adedoyin (2020) [36] examined whether the effects of EPU on carbon emissions are established in China by employing ARDL estimation based on the annual data from 1970 to 2018, and concluded that EPU cannot change the carbon emissions in China, which may be attributed to its sustainable corporate policies. Similarly, Liu and Zhang (2021) [39] offered a similar idea for the central and western region of China. On the other hand, Adedoyin and Zakari (2020) [36] proposed that the influence of EPU on CO_2_ emissions is not constant short-term and long-term; specifically, while EPU reduces CO_2_ emissions initially, it would increase the CO_2_ emissions long-term. Similarly, Anser et al., (2021) [40] investigated the short-term and long-term influences of EPU on carbon emissions, by utilizing the data from 1990 to 2015 for the 10 largest carbon-emitting countries via PMG-ARDL estimation; the empirical results, by employing the indicator of WUI to measure EPU, supported that increasing EPU would mitigate the CO_2_ emissions in the short run while it would escalates CO_2_ emissions in the long run. 

Aside from utilizing the variable of CO_2_ emissions to measure the environmental degradation, Anser et al. (2021) [40] captured environmental performance by ecological footprint, to empirically investigate the role of EPU in environmental performance; they argued that higher EPU would improve the ecological footprint, meaning that EPU benefits environmental performance. 

Given the above review of the relevant literature, we can conclude that scant consideration given to the impact of EPU on total environment, since most studies simply captured environmental damage by carbon emissions; Furthermore, the conclusion for the influence of EPU on CO_2_ emissions is not inconclusive among existing literature, and needs to be further investigated by using data for multinational countries and an appropriate model. In addition, existing literature often utilizes the EPU index to measure EPU, which ignores political uncertainty and has no common base for different countries. Thus, our study tries to bridge the gap between existing studies.

## 3. Variables, Data and Methodology

### 3.1. Variables

Environmental Performance Index(EPI): previous studies often utilize variables such as CO_2_ emissions, greenhouse gas emissions, ecological footprint, or environmental performance index (EPI) to measure environmental performance (Wang et al., 2021b; Atsu and Adams, 2021) [8,29]. However, these measurements have been doubted, since they only cover some aspects of environmental damage, ignoring other aspects such as forest, water, soil, etc. Unlike such variables, EPI comprehensively captures two categories of variable: total environmental damage, which covers environmental health and ecosystem vitality, air quality, sanitation and drinking, water, heavy metals, waste management and so on; and ecosystem vitality, which covers biodiversity and habitat, ecosystem services, fisheries, climate change, pollution emissions, agriculture and water resources (Wen et al., 2016) [41]. Based on such advantages of EPI, we also utilized this index to capture total environmental performance (denoted by EPI), which was given by the Yale Center for Environmental Law and Policy and the Center for International Earth Science Information Network at Columbia University. 

Economic policy uncertainty (Uncertainty): To gain a general conclusion on the role of EPU in environmental performance worldwide, we utilized the data of WUI to measure EPU in line with Adams et al. (2020) [23]. Specifically, Ahir et al. (2018) [32] construct quarterly indices of EPU by using frequency counts of “uncertainty” (and its variants) in the quarterly Economist Intelligence Unit (EIU) country reports, which are scaled by the total number of words in each report and multiplied by 1000, to make them comparable worldwide. Since the EPI data are year-frequency, they take the mean value of quarterly WUI to measure EPU on year-frequency (Atsu and Adams, 2021) [29], which is denoted by Uncertainty. A higher value of Uncertainty usually stands for a higher EPU. 

According to previous articles, the focus on environmental performance and the extended STIRPAT model (Wen et al., 2016; Wang et al., 2021b) [8,41], we include the following variables in our model: the economic performance (GDP) (which stands for Affluence in the STIRPAT model), industrial share (IND), population (POP) (representing Population in the STIRPAT model), density of people (Density), structure of population (Aging), process of urbanization (Urban), international trade (Trade), utilization of foreign investment (IFDI), and green technologies (GI) (representing Population in the STIRPAT model). The definition and source of such variables can be seen in Table 1.

### 3.2. Data

The EPI data for some countries were sourced from the Yale Center for Environmental Law, and those for other countries were obtained from the Policy and Center for International Earth Science Information Network at Columbia University (Website of the Yale Center for Environmental Law and Policy: https://epi.envirocenter.yale.edu/epi-downloads, accessed on 14 May 2021. Website of the Center for International Earth Science Information Network: https://sedac.ciesin.columbia.edu/data/collection/epi, accessed on 21 May 2021). The Uncertainty data were provided by Ahir et al. (2018) [32] (http://www.policyuncertainty.com/wui_quarterly.html, accessed on 3 June 2021). The GI data were obtained from OECD statistics (2019), while those for the remaining variables were provided by World Development Indicators (http://databank.worldbank.org/data/reports.aspx?source=wdi-database-archives-(beta), accessed on 5 February 2021). We merged all of the data together according to dimensions such as country and year, and filtered the observations by following such principles as dropping the observations with missing values. We finally constructed a panel dataset with 2325 observations for 137 countries during 2001 to 2018. Similarly to Wang et al. (2021b) [8], we took the log for these variables.

We provide a description of the variables in Table 2. It can be seen that the minimum, mean, median and maximum for EPI are 2.843, 4.074, 4.148 and 4.522, respectively; while its standard deviation (S.D), Skewness and Kurtosis are 0.315, −0.858 and 3.461, respectively; this implies that there exists no big difference in environmental performance among such countries. While we analyze the distribution of EPU, we can find that the minimum, and maximum for Uncertainty are 0, and 0.851, respectively, while its S.D is 0.119. For the other variables, taking GI as an example, it is obvious that its S.D is 2.304, while its minimum and maximum are 0 and 8.89, indicating that the green technologies fluctuate more among these sample countries. 

### 3.3. Estimating Methods

According to previous literature, such as Wen et al., (2016) [41] and Wang et al., (2021b) [8], environmental performance is not only influenced by current situations, but also by previous environmental performance. To include the dynamic process in our estimation, we chose the generalized method of moment to conduct an empirical investigation. However, as suggested by Wang et al., (2019) [42], the system GMM estimation has some advantages over difference GMM estimations, so we finally utilized the system GMM estimation to conduct our empirical investigation, which is given as following.

(1) $$EPI_{i,t}=\alpha_{1}EPI_{i,t-1}+\theta Uncertainty_{it}+\beta^{\prime }X+u_{i}+u_{t}+\varepsilon_{it},$$

where i=1,2,3…N , stands for the individual country, whereas t=1,2,3…T, represents the dimension of year. EPI is the dependent variable, while $EPI_{i,t-1}$ is the first lag of it, Uncertainty is the variable of EPU. *X* represents the control variables, β represents the vector terms for the corresponding coefficients, and $u_{i}$ and $u_{t}$ stand for the fixed effect of individual country and year, respectively. 

## 4. Estimating Results

### 4.1. Baseline Results

Following previous articles, we only took the EPU and time effects into account at first, hence taking other control variables into account thereafter. Their results are given in Table 3. In column (1), the coefficient of uncertainty is −0.372, which is significantly negative at 1%, indicating that EPU would inhibit the environmental performance. When we take other variables and the time-fixed effect into account, the coefficient of uncertainty in columns (2)–(4) is −0.046, −0.046 and −0.191, respectively; these are all significantly negative at 1%, again confirming the idea that EPU negatively affects EPI. Aside from this, we also conclude that the coefficient of L. EPI is 0.814, which is significantly positive at 1%, indicating that environmental performance is positively affected by previous level of environmental performance. the remaining results in columns (2)–(4) also offer similar results.

These GMM estimating results strongly support that EPU exerts a negative influence on environmental performance; this supports previous works of literature that state that EPU would bring about more CO_2_ emissions, such as Adams et al., (2020) [23]. 

### 4.2. Increasing EPU

During the post-coronavirus era, the international situation has become tense and EPU is growing (Wang et al., 2021b) [8]. Given this external circumstance, it is necessary to investigate whether a quick change in EPU would have an effect on environmental performance, which can more comprehensively uncover the exact role of EPU in environmental performance. To investigate this issue, we set four variables—Δuncertainty, Δuncertainty_trade, Δuncertainty_absolute and Δuncertainty_season—representing the differences between the earlier four variables for EPU. Having utilized these four new variables to capture the change of EPU, the estimating results are given in Table 4. We find that the coefficient of Δ*uncertainty* in column (1) is −0.288, which is significantly negative at 1%, suggesting that the increase in EPU would result in worse environmental quality. The other results in column (2)–(4) also support such idea. 

### 4.3. The Role of Social Development in EPU’s Impact on Environmental Performance

Our earlier results confirm that EPU would negatively affect environmental performance; however, the role of EPU on environmental performance may depend on other social circumstances. To examine the specific influence of EPU on the environment in different fundamental countries, we included the cross term of EPU and other social variables, such as economic development, economic performance, globalization, trade openness and the quality of governance, to test the moderating effect of such social variables. 

To begin with, we set the cross term of uncertainty*OECD to study the moderating effect of economic development in EPU’s influence on environmental performance, which can be seen in column (1) of Table 5 (*OECD* is a binary variable denoting if the countries belong to the Organization for Economic Cooperation and Development, thus OECD = 1; otherwise, 0. Compared to non-OECD countries, OECD countries express a higher level of economic development). It is obvious that the coefficient of uncertainty*OECD is 0.226, which is statistically significant and positive at 10%. However, the coefficient of *uncertainty* is −0.254, significantly negative at 1%, and the absolute value for the coefficient of uncertainty*OECD is less than that for *uncertainty*, implying that EPU would negatively affect environmental performance. However, OECD countries would reduce the negative influence of EPU on environmental performance. This may be because the environmental policies and regulations of OECD countries are more advanced than those of non-OECD countries; the complete legal system and mature markets would reduce the external shock and confusion brought by EPU, thus weakening the negative effect of EPU on the environment.

Hence, we examined the moderating effect of economic performance on EPU’s impact on environmental performance, by setting the interactive term of uncertainty*GDP, which can be seen in column (2). The coefficient of uncertainty*GDP is 0.076, which is significantly positive at 1%, suggesting that better economic performance would contribute to the reduction in the negative effect of EPU on environmental performance. This is mainly because better economic performance offers governments more power to deal with EPU; moreover, even under serious EPU, better economic performance can make it is possible for governments to allocate some fiscal expenditure to environmental protection, thus reducing the adverse environmental effects brought by EPU (Liu and Zhang, 2021) [39]. Aside from this, according to the environmental Kuznets relationship, pollution levels eventually fall as income increases. 

Furthermore, we examined the question of whether globalization can affect the negative influence of EPU on environmental performance, by setting the cross term of uncertainty*GLOBAL (since globalization is a multifaceted phenomenon with essential economic, social and political dimensions, we utilized the globalization data provided by KOF Swiss Economic Institute (2020) to measure the country’s globalization, denoted by *Global*). According to the result of column (3) in Table 4, the coefficient of uncertainty*GLOBAL is 0.430, which passes the significance test at 5%, meaning that higher globalization can help to weaken the negative influence of EPU on environmental performance. This is mainly because globalization would promote environmental globalization, and give domestic countries more of a chance to interact with other countries, which may help governments to deal with the shock caused by EPU; aside from this, the effects of technology, environmental awareness and NGOs focusing on environmental protection led by globalization, would efficiently reduce the negative influence of EPU on environmental performance. Moreover, when a country sells to customers in other countries, the environmental standards of the buyers in countries with high environmental standards influence the practices of the sellers, in addition to any influence from environmental standards in the sellers’ countries.

At last, we also studied whether the quality of governance can change role of EPU in environmental performance by employing the cross term of uncertainty*WGI, whose result is given by column (4) (We measured the quality of governance by utilizing the data provided by World Governance Index, denoted by *WGI*). We can see that the coefficient of uncertainty*WGI is 0.427, significantly positive at 10%, indicating that better quality of governance can effectively reduce the negative effect of EPU on environmental performance. It is worth noting that the absolute value of the coefficient for uncertainty*WGI is larger than that of *uncertainty* in column (4). The potential reason for this may be that a better quality of governance is critical to regulating activities with adverse environmental effects. Even with the growing of EPU, the regulations determined by better governance would contribute to the protection of the environment, meaning that better quality of governance would reduce the decrease in environmental performance caused by EPU. 

### 4.4. The Role of Political Regimes in EPU’s Impact on Environmental Performance

Aside from abovementioned factors, political regimes also determine the power of governments when they face external shock such as EPU. Additionally, Wen et al., (2016) [41] suggested that environmental performance is influenced by the quality of institutions and policies. The influence of EPU on environmental performance probably also varies between different political regimes. Moreover, with the worldwide growth of EPU and environmental pollution known as a global issue, the international aspect of democracy is beneficial for countries in dealing with external shock such as EPU, and promoting the development of international NGOs or treaties to deal with environmental problems (Winslow, 2005) [43]. To comprehensively study whether political regimes determine the role of EPU in environmental performance, we captured the difference among political regimes by utilizing the data for democracy and political ideology. 

Firstly, we tested the difference in EPU’s influence on environmental performance between left-wing and right-wing governments; specifically, we divided the whole sample into two sub-samples according to the political ideology of the ruling parties—one was the left-wing sub-sample, and the other was the non-leftist sub-sample. In line with Chang et al., (2015) [44], if the chief executive party was communist, socialist, social democratic, or left-wing, those countries belonged to the left-wing sub-sample; otherwise, they were classified as non-leftist (the data for political ideology were derived from DPI (2019), in line with Wang et al., (2019) [42]). The results based on these two samples are given in the columns (1) and (2) of Table 6, respectively. The coefficient of *Uncertainty* in column (1) is −0.055, which is significantly negative at 1%, offering the conclusion that EPU usually leads to worse environmental performance among leftist countries. Similarly, the result in column (2) shows that the coefficient of *Uncertainty* is −0.069, significantly negative at 1%, meaning that in rightist countries, EPU also negatively affects environmental performance. Comparing the coefficient of *Uncertainty* in columns (1) and (2), we can see that the coefficient of *Uncertainty* in column (2) is less than that in column (1), suggesting that the negative influence of EPU on environmental performance is weaker among left-wing countries. The potential reason for this is that, compared to right-wing parties, left-wing parties care more about environmental quality, since their supporters are almost all blue collar or low-income citizens who cannot afford the professional equipment to adapt to or mitigate environmental damage; thus the left-wing governments prefer to carry out more measures to protect the natural environment, even though EPU increased; thus a lower influence of EPU on environmental performance is brought about among leftist countries. 

We further studied the question of whether the democratic or autocratic regimes can also change EPU’s impact on environmental performance by dividing the whole sample into two sub-samples—Democracy and Non-democracy—according to the dummy variable of democracy provided by Cheibub et al. (2010) [45] originally, and extended by Bjørnskov and Rode (2019) [46]. We then empirically tested EPU’s influence on environmental performance by utilizing these two sub-samples, which can be seen in columns (3) and (4). The coefficient of *Uncertainty* in column (3) is −0.061, which is significantly negative at 1%, implying that EPU can negatively affect environmental performance among democracies. Similarly, the results in column (4) show that the coefficient of *Uncertainty* is −0.149, which is significantly negative at the 1% level, suggesting that EPU would negatively affect environmental performance among non-democracies. According to the results in column (3) and (4), we can conclude that that the negative influence of EPU on environmental performance is stronger among non-democracies than in democracies. The potential reason for this is that, compared to non-democracies, democracies are somewhat at an advantage in dealing with uncertainty thanks to, for example, the appropriate policies and short time-lag for implementing measures; thus, the negative effect of uncertainty on environmental performance is reduced in democracies. Furthermore, the public’s understanding of environmental protection and citizens’ pressure on governments are higher among democracies, thus making the governments attach more importance to environmental protection, which may reduce the negative influence of uncertainty on environmental protection. 

## 5. Conclusions and Policy Implications

With the growth of economic policy uncertainty (EPU) worldwide and the importance of environmental protection, this study mainly pays attention to the influence of EPU on total environmental performance. To investigate this issue, we collected cross-country data for 137 countries from 2001 to 2018 to carry out system GMM estimation. The baseline result confirms that EPU does some harm to the total environment, which may be attributed to the reduction in activities related to environmental protection caused by EPU; this was also confirmed when we utilized new measurements of EPU or new samples, removing the outliers to conduct robustness tests. Moreover, the increase in EPU would also negatively affect environmental performance. In addition, in examining the moderating effect of other factors in EPU’s influence on environmental performance, we see that the negative effect of EPU on environmental performance among OECD countries is lower than that in non-OECD countries, suggesting that a higher level of economic development would reduce the adverse environmental effect led by EPU. In addition, higher globalization and more international trade would weaken the negative effect of EPU on environmental performance, and better governance would reduce EPU’s influence on environmental performance. Aside from these, we also tested whether the role of EPU in environmental performance varies among different political regimes, the results support that the negative influence of EPU on globalization is stronger in autocracies than in democracies, whereas a left-wing government can somewhat reduce EPU’s influence on environmental performance. 

According to our empirical findings, we offer following policy implications for policymakers to improve environmental performance. Firstly, given that EPU would have a negative influence on environmental performance, governments can spare more effort to protect the stability and predictability of economic policies, especially for environmentally friendly policies; if they prefer to conduct new policies, the transition should be smooth, especially during periods of election or when experiencing a big external shock such as coronavirus, the stability of economic policies can not only spur on economic incentive, but also contribute to the improvement of the environmental performance, which are both essential for national sustainable development. Secondly, regarding the specific dimension of EPU, trade uncertainty also negatively affects environmental performance. During the post coronavirus era, the uncertainty of trade also experienced some increase. To reduce the negative effects of trade uncertainty, policymakers should continue their earlier preference of international trade or trade between domestic countries, as well as enact long-term policies to keep sustainable policies for trade activities, which is beneficial for environmental protection. 

In addition, the results for moderating effect show that uncertainty’s negative effect on environmental performance is higher among emerging markets. For such countries, they should make more effort to build better and more complete policy systems, which can be learned from OECD countries, to improve their ability to deal with external or internal shock and reduce EPU; Additionally, while they try to improve economic performance, a long-term plan to protect the natural environment should be enacted as well, which is essential to weaken the negative influence of EPU on environmental performance. Moreover, the results of the moderating effect of globalization suggest that a higher level of globalization would reduce the uncertainty’s negative influence on environmental performance. With the growing of protectionism and slowing of globalization, countries should be aware of the importance of globalization in facing uncertainty and environmental concerns. Finally, better governance has the benefits of reducing the negative effect of EPU on environmental performance, brought about by the turbulent international environment and post-novel coronavirus. Countries which prefer to can improve environmental performance by improving the quality of national governance, such as by gaining better control of corruption or protection of property rights, which can help countries to gain better environmental performance.

Due to our limited time, one may further explore the effect of the measures of freedom on environmental quality. One could also consider alternative measures of policy uncertainty and effectiveness, such as the adherence to particular standards, the size of fines against particular infractions, or actual levels of emissions per capita.

## Figures and Tables

**Table 1:** Variable definitions.

Variable	Definition	Source
Dependent variables		
EPI	The score of environmental performance	YCELP, CIESIN
Independent Variables		
Uncertainty	The score of economic policy uncertainty	Ahir et al. (2018) [32]
Control variables		
GDP	real GDP per capita which is constant in 2011 US dollars	WDI (2020)
IND	The proportion of industrial value added to GDP.	WDI (2020)
POP	The number of the total population	WDI (2020)
Density	The number of people per square km	WDI (2020)
Aging	Proportion of people aged 65 or above in total population	WDI (2020)
Urban	The share of urban residentials in total population	WDI (2020)
Trade	Share of exports and imports to GDP	WDI (2020)
IFDI	Ratio of IFDI to GDP	WDI (2020)
GI	The number of patent applications about environmental management	OECD (2020)

**Table 2:** Summary of descriptive statistics.

Variable	N	Mean	Min	Median	Max	S.D	Skewness	Kurtosis
*EPI*	2325	4.074	2.843	4.148	4.522	0.315	−0.858	3.461
*Uncertainty*	2325	0.159	0.000	0.133	0.851	0.119	1.192	5.017
*GDP*	2325	8.467	5.356	8.433	11.431	1.498	0.107	1.974
*IND*	2325	3.304	0.835	3.289	4.486	0.380	−0.532	6.932
*POP*	2325	16.525	14.045	16.248	21.055	1.342	0.731	3.487
*Density*	2325	4.167	0.939	4.310	8.981	1.278	−0.023	3.726
*Aging*	2325	2.021	0.522	1.909	3.353	0.621	0.200	1.829
*Urban*	2325	3.984	2.247	4.083	4.615	0.461	−1.056	3.632
*Trade*	2325	4.278	0.155	4.286	6.083	0.535	−1.494	14.626
*IFDI*	2325	1.405	0.000	1.356	4.648	0.754	0.649	3.837
*GI*	2325	1.937	0.000	0.916	8.890	2.304	1.096	3.256

**Table 3:** SYS-GMM estimations for EPU’s impact on EPI.

	(1)	(2)	(3)	(4)
L. EPI	0.814 ***	0.665 ***	0.454 ***	0.363 ***
	(155.008)	(99.538)	(62.360)	(12.932)
Uncertainty	−0.372 ***	−0.046 ***	−0.046 ***	−0.191 ***
	(−18.510)	(−3.334)	(−2.905)	(−3.245)
GDP		0.016	0.156 ***	0.203 ***
		(1.506)	(9.526)	(3.976)
IND		0.194 ***	0.013	−0.049
		(11.572)	(0.915)	(−1.418)
POP			−10.291 ***	−3.608
			(−4.491)	(−0.766)
Density			11.532 ***	5.293
			(4.766)	(1.100)
Aging			−0.062	0.607 ***
			(−0.846)	(3.313)
Urban				−0.314
				(−1.367)
Trade				0.011 *
				(1.684)
IFDI				−0.016 *
				(−1.669)
GI				−0.136 ***
				(−6.789)
Year FE	no	yes	yes	yes
N	2051	2051	2051	2051
AR (1)	−8.619	−7.376	−5.931	−6.311
AR (1)-P	0.000	0.000	0.000	0.000
AR (2)	−0.970	−1.057	1.449	−1.536
AR (2)-P	0.332	0.291	0.147	0.125
Hansen-P	0.310	0.162	0.225	0.497
Wald	376.14	416.98	546. 91	578.34

Notes: ***, and * indicate statistical significance at the 1%, and 10% levels, respectively. Z-statistics are in parenthesis.

**Table 4:** Robustness test—increasing or slowing EPU.

	(1)	(2)	(3)	(4)
L.EPI	0.398 ***	0.373 ***	0.413 ***	0.371 ***
	(12.960)	(13.938)	(13.340)	(12.488)
ΔUncertainty	−0.288 ***			
	(−5.810)			
ΔUncertainty_trade		−0.009 ***		
		(−3.557)		
ΔUncertainty_absolute			−0.028 ***	
			(−4.685)	
ΔUncertainty_season				−0.258 ***
				(−5.194)
GDP	0.175 ***	0.206 ***	0.159 ***	0.210 ***
	(3.019)	(5.074)	(2.814)	(3.557)
IND	−0.042	−0.027	−0.003	−0.036
	(−1.038)	(−0.913)	(−0.073)	(−0.888)
POP	−3.904	−5.062	−0.935	−1.970
	(−0.817)	(−1.188)	(−0.185)	(−0.355)
Density	5.500	6.631	2.405	3.687
	(1.114)	(1.523)	(0.461)	(0.644)
Aging	0.594 ***	0.453 ***	0.596 ***	0.652 ***
	(3.433)	(2.689)	(3.527)	(3.518)
Urban	−0.300	−0.411 *	−0.280	−0.461 *
	(−1.296)	(−1.862)	(−1.132)	(−1.916)
Trade	0.016 **	0.003	0.016 **	0.017 **
	(2.273)	(0.671)	(2.416)	(2.461)
IFDI	−0.019 *	−0.010	−0.022 **	−0.016
	(−1.798)	(−1.222)	(−2.265)	(−1.427)
GI	−0.139 ***	−0.112 ***	−0.128 ***	−0.144 ***
	(−5.856)	(−5.569)	(−5.553)	(−6.312)
Year FE	yes	yes	yes	yes
N	2051	2051	2051	2051
AR (1)	−6.673	−6.340	−6.455	−6.611
AR (1)-P	0.000	0.000	0.000	0.000
AR (2)	−0.471	−1.376	−0.776	−1.267
AR (2)-P	0.637	0.169	0.438	0.205
Hansen-P	0.426	0.511	0.534	0.497
Wald	943.08	716.28	749.32	649.33

Notes: ***, **, and * indicate statistical significance at the 1%, 5%, and 10% levels, respectively. Z-statistics are in parenthesis.

**Table 5:** Moderating effect of social development.

	(1) OECD	(2) GDP	(3) GLOBAL	(4) WGI
L.EPI	0.391 ***	0.389 ***	0.352 ***	0.407 ***
	(14.297)	(13.751)	(12.033)	(13.734)
Uncertainty	−0.254 ***	−0.816 ***	−1.991 **	−0.424 ***
	(−3.961)	(−4.025)	(−2.265)	(−3.776)
Uncertainty*OECD	0.226 *			
	(1.847)			
Uncertainty*GDP		0.076 ***		
		(2.817)		
Uncertainty*GLOBAL			0.430 **	
			(2.017)	
GLOBAL			1.391 ***	
			(4.169)	
Uncertainty*WGI				0.427 *
				(1.943)
WGI				1.404 ***
				(5.403)
GDP	0.211 ***	0.211 ***	0.022	0.045
	(3.632)	(3.558)	(0.457)	(0.718)
IND	−0.046	−0.047	−0.057 *	−0.014
	(−1.259)	(−1.310)	(−1.678)	(−0.327)
POP	−4.305	−2.715	−1.738	−1.643
	(−0.970)	(−0.610)	(−0.464)	(−0.387)
Density	6.218	4.546	2.780	3.326
	(1.382)	(1.006)	(0.732)	(0.766)
Aging	0.654 ***	0.636 ***	0.427 ***	0.665 ***
	(3.275)	(3.257)	(2.690)	(3.598)
Urban	−0.376 *	−0.307	−0.075	−0.175
	(−1.742)	(−1.455)	(−0.329)	(−0.759)
Trade	0.012 *	0.012 **	0.011	0.011 *
	(1.883)	(2.046)	(0.491)	(1.814)
IFDI	−0.020 *	−0.017 *	−0.025 **	−0.014
	(−1.917)	(−1.693)	(−2.517)	(−1.420)
GI	−0.134 ***	−0.132 ***	−0.098 ***	−0.128 ***
	(−6.353)	(−6.314)	(−5.549)	(−6.301)
Year FE	yes	yes	yes	yes
N	2051	2051	2035	2035
AR (1)	−6.182	−6.252	−5.976	−6.380
AR (1)-P	0.000	0.000	0.000	0.000
AR (2)	−1.236	−1.240	−2.277	−1.240
AR (2)-P	0.217	0.215	0.023	0.215
Hansen-P	0.449	0.446	0.325	0.350
Wald	346.19	498.26	691.57	711.95

Notes: ***, **, and * indicate statistical significance at the 1%, 5%, and 10% levels, respectively. Z-statistics are in parenthesis.

**Table 6:** Interactive effect of political regimes and governmental ideology.

	(1) Left	(2) Right	(3) Democracy	(4) Non-Democracy
L. EPI	0.224 ***	0.203 ***	0.176 ***	0.317 ***
	(11.812)	(13.249)	(8.110)	(17.557)
Uncertainty	−0.055 ***	−0.069 ***	−0.061 ***	−0.149 ***
	(−2.857)	(−4.517)	(−3.027)	(−7.365)
GDP	0.168 ***	0.009	−0.110 ***	0.084 **
	(6.017)	(0.254)	(−4.660)	(2.225)
IND	−0.047 ***	0.113 **	−0.034 ***	−0.000
	(−4.391)	(2.486)	(−4.300)	(−0.017)
POP	−2.116	13.783 ***	−4.937 ***	−3.466
	(−1.418)	(4.445)	(−3.407)	(−1.603)
Density	2.624 *	−12.318 ***	4.097 ***	5.257 **
	(1.835)	(−3.957)	(2.729)	(2.378)
Aging	−0.108	0.250	−0.387 ***	0.362 ***
	(−1.054)	(1.281)	(−5.111)	(3.702)
Urban	0.037	−0.160	0.640 ***	−0.205
	(0.253)	(−0.619)	(4.420)	(−1.254)
Trade	0.063 ***	−0.050 ***	−0.002	0.006
	(6.563)	(−2.676)	(−0.855)	(0.354)
IFDI	−0.011 ***	0.028 ***	0.004	0.031 ***
	(−5.519)	(4.019)	(1.094)	(4.397)
GI	−0.011 ***	−0.003	−0.036 ***	−0.080 ***
	(−2.768)	(−0.555)	(−8.976)	(−8.883)
Year FE	yes	yes	yes	yes
N	576	551	786	1233
AR (1)	−3.365	−2.189	−2.968	−5.636
AR (1)-P	0.001	0.029	0.003	0.000
AR (2)	−1.768	1.561	−0.067	−1.209
AR (2)-P	0.177	0.119	0.947	0.227
Hansen-P	0.981	0.915	0.996	0.709
Wald	461.34	591.48	667.31	430.28

Notes: ***, **, and * indicate statistical significance at the 1%, 5%, and 10% levels, respectively. Z-statistics are in parenthesis.

## Data Availability

The data used to support the findings of this study are available from the corresponding author upon request.
